# Supplementary material for: A Rapid and Sensitive Aptamer-Based Biosensor for Amnesic Shellfish Toxin Domoic Acid
Source: Bioengineering (Basel). 2022 Nov 11;9(11):684. doi: 10.3390/bioengineering9110684 (PMC9687209; doi:10.3390/bioengineering9110684)
Supplement: Supplementary file 1 [file bioengineering-09-00684-s001.zip › bioengineering-1988287-supplementary.pdf]

Supplementary Materials

A rapid and sensitive aptamer-based biosensor for amnesic shellfish toxin domoic acid

Luming Zhao [1,†], Han Guo [1,†], Han Chen [1,†], Bin Zou [1], Chengfang Yang [1], Xiaojuan Zhang [2], Yun Gao [1], Mingjuan Sun [1,*] and Lianghua Wang [1,*]

[1] Department of Biochemistry and Molecular Biology, College of Basic Medical Sciences, Naval Medical University, Shanghai 200433, China

[2] College of Medicine, Shaoxing University, 900th Chengnan Avenue, Shaoxing 312000, China

* Correspondence: sunmj@smmu.edu.cn (M.S.); lhwang@smmu.edu.cn (L.W.)

† These authors contributed equally to this work.

1. Analysis Software and Methods

1.1. Sequence Analysis of Aptamers

Sequences from high throughput sequencing and cloning sequencing were analyzed by Clustal X 2.1 for multiple sequence alignment. Sequences were clustered and divided into several families.

The secondary structure and Gibbs free energy of candidate aptamers were predicted by the web server of mfold (http://www.unafold.org/mfold/applications/dna-folding-form.php). The folding temperature was set to 25 ˚C, and the concentrations of Na^+ and Mg^{2+} were set to 177.7 mM and 0.492 mM, respectively. Other parameters were set to be the default in mfold.

1.2. 3D Structure Modeling of Aptamer

The secondary structure of aptamer was obtained by RNAfold web server. The sequence of aptamer was submitted in RNAfold, a web server predicting secondary structures of single stranded corresponding RNA or DNA sequences. RNAfold output the optimal secondary structure in dot-bracket notation with a thermodynamic ensemble free energy. The 3D structure of corresponding RNA was obtained by submitting the secondary structure and sequence on RNA Denovo (https://new.rosettacommons.org/docs/latest/application_ documentation/rna/rna-denovo-setup). The RNA Denovo was implemented by Rosetta, which can be carried out reasonably automatically prediction of large RNA 3D structures. Finally, the corresponding RNA 3D structure was converted into DNA 3D structure of aptamer using AMBER16.

1.3. Molecular Docking

Molecular docking of DA and aptamer was performed by MOE Dock. 2D structures of molecules were downloaded from PubChem and transformed to 3D structures in MOE by energy minimization, as ligands. Before docking, the force field of AMBER10: EHT and the implicit solvation model of Reaction Field (R-field) was selected. The docking process adopted the flexible "induced fit" mode, and the side chains of the combined pocket bases can be optimized and adjusted according to the ligand conformation, and the weight to constrain the rotation of the side chain was set to 10. Firstly, the binding modes of ligands were first ranked by the London dG scoring function, and the first 30 conformations were further optimized, and the binding free energies were re-evaluated by the GBVI/WSA dG method. The binding mode with the lowest free energy was chosen as the best binding mode. Diagrams of intermolecular binding patterns were completed on PyMOL (www.pymol.org).

1.4. Molecular Dynamics (MD) Simulation

After docking, the DA-aptamer complex was optimized by MD simulation. Sodium/chloride counterbalance ions were added to the complex to neutralize the system and solvated in a rectangular water box of TIP3P to form a 10 Å solvent layer between the edge of the water box and the solute surface.

All molecular dynamics simulations were performed by AMBER16 software. AMBER GAFF and FF14SB force fields were applied, and all hydrogen atoms involved in covalent bonds were restricted using the SHAKE algorithm with a time step of 2 fs. The Particle mesh Ewald (PME) method was used to deal with the long-range electrostatic interactions. Two minimization steps were performed for each solvation system before the heating step. The initial 4000 minimization cycles were performed with all heavy atoms confined by50 kcal/(mol·Å2), while solvent molecules and hydrogen atoms were free to move. Unconstrained minimization is then performed, including 2000 steepest descent minimization cycles and 2000 conjugate gradient minimization cycles. The entire system was then heated first from 0 K to 300 K at 100 ps at a constant volume using Langevin dynamics and then equilibated for 150 ps at a constant pressure of 1 atm. Periodic boundary dynamics simulations were performed for the entire

system, where the NPT (constant components, pressure, and temperature) ensemble was performed at a constant pressure of 1 atm and 300 K during the production step. In the equilibration phase, 100 ns was performed in simulations.

1.5. Data analysis of BLI assay

Data analysis of BLI assay was performed by ForteBio Data Analysis 11.0 Software (Sartorius, Shanghai, China). The affinity constant K_D, association constant K_{on}, and dissociation constant K_{dis} were detected to explore the binding affinity of aptamer and target. The constant value was fitted by 1:1 binding model. When $R^2 > 0.8$ and $X^2 < 3$, the data is valid.

2. Statistical Analysis

Statistical analysis was performed by Microsoft Excel 16.65 and GraphPad Prism 8.4.3 Software. One way ANOVA was used to analyze the variance and Dunnett's t-test was used to compare the significance between all groups for the various experiments. The probabilities were $p < 0.05$ (*), $p < 0.01$ (**).

3. Figures and Tables

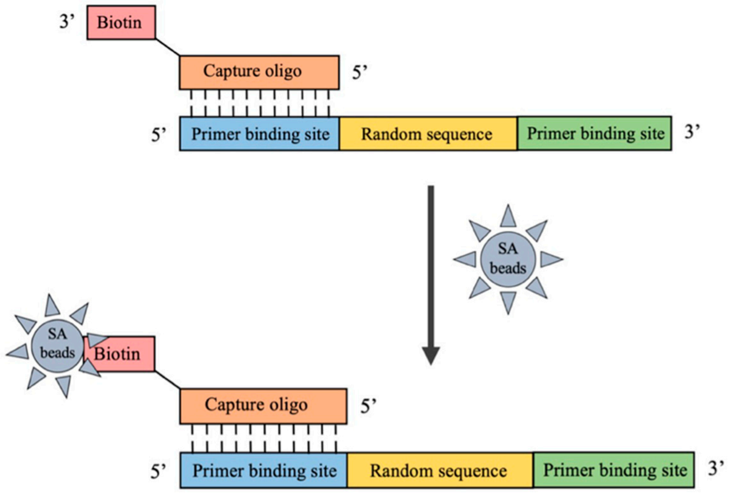

Figure S1. Immobilization of the ssDNA library. The ssDNA and capture oligo were hybridized by base pairing, then the hybrid was immobilized on beads through the interaction between streptavidin and biotin.

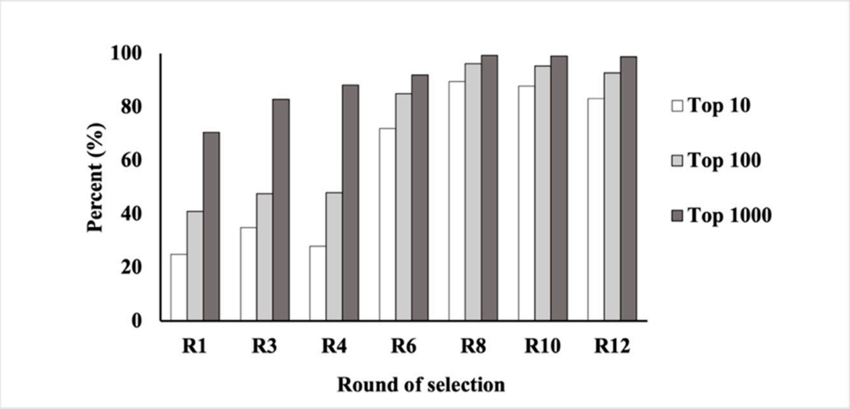

Figure S2. The abundance of top 10, top 100, top 1000 sequences in the whole sequencing results of different selection rounds.

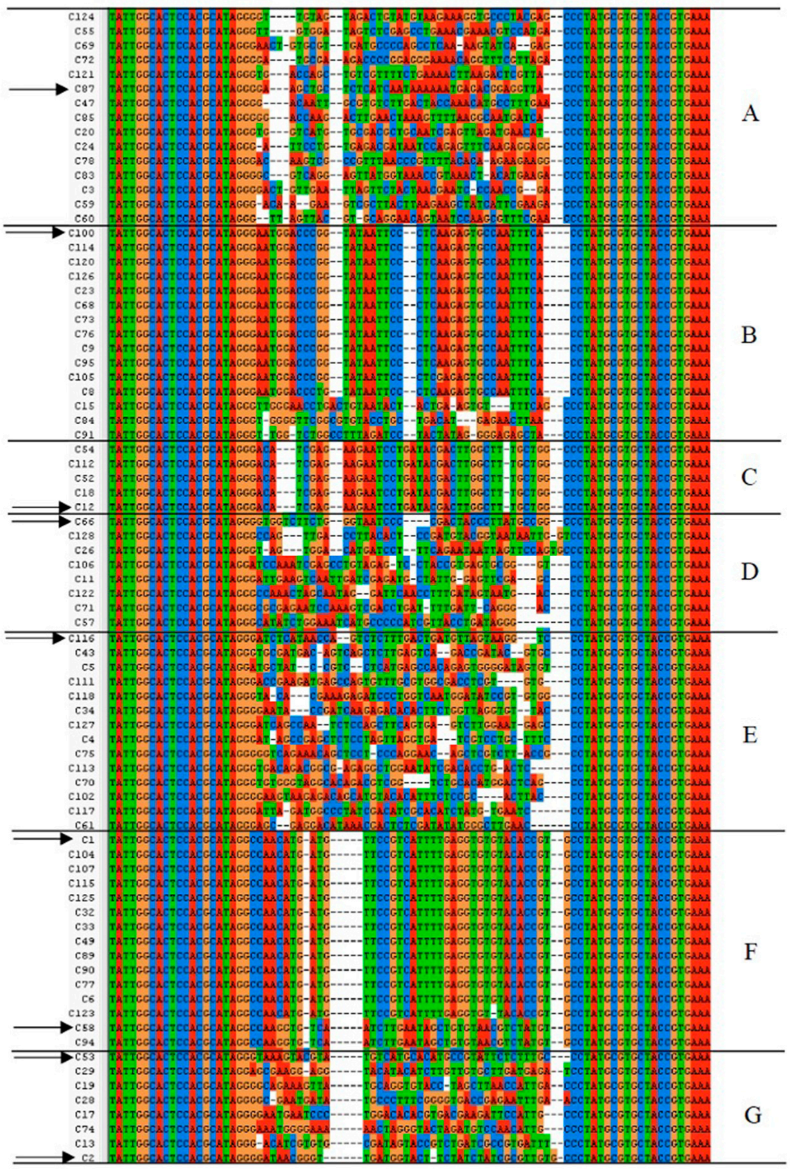

Figure S3. Multiple sequence alignment of sequences from cloning sequencing by Clustal X 2.1. These sequences were grouped based on conservation into 7 families (A-G), and a representative sequence was chosen from each group for further analysis (C87, C100, C12, C66, C116, C1, C58, C53 and C2).

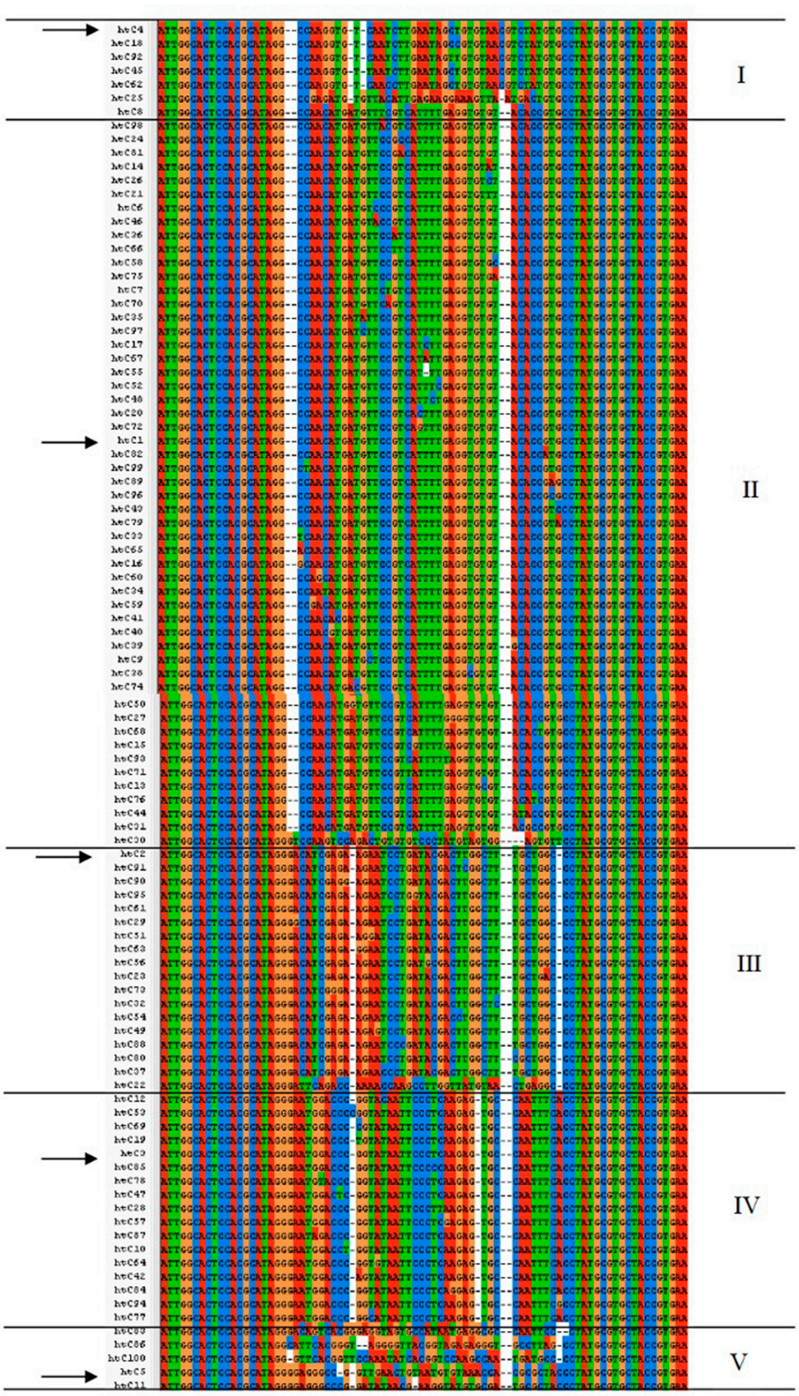

Figure S4. Multiple sequence alignment of sequences from high-throughput sequencing (HST) by Clustal X 2.1. These sequences were grouped based on conservation into 5 families (I-V), and a representative sequence was chosen from each group for further analysis (htC1, htC2, htC3, htC4, htC5).

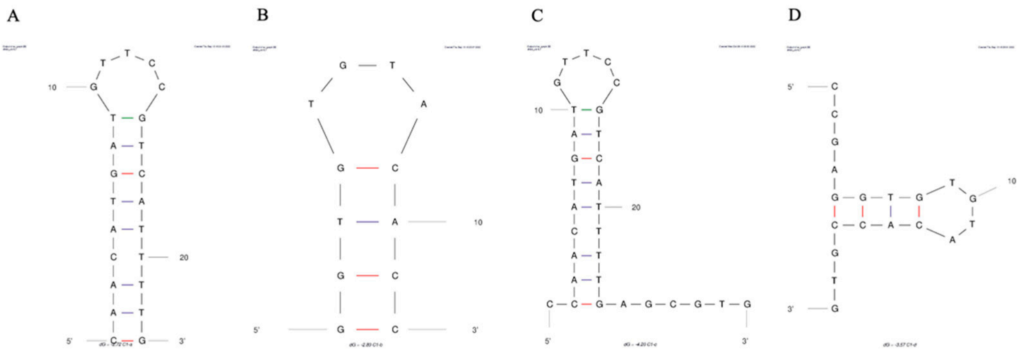

Figure S5. Secondary structure prediction of aptamer C1-a (**A**), aptamer C1-b (**B**), aptamer C1-c (**C**), and aptamer C1-d (**D**) with their respective lowest Gibbs free energy value by mfold program. The folding temperature is 25 ˚C, and the concentrations of Na^+ and Mg^{2+} were 177.7 mM and 0.492 mM, respectively.

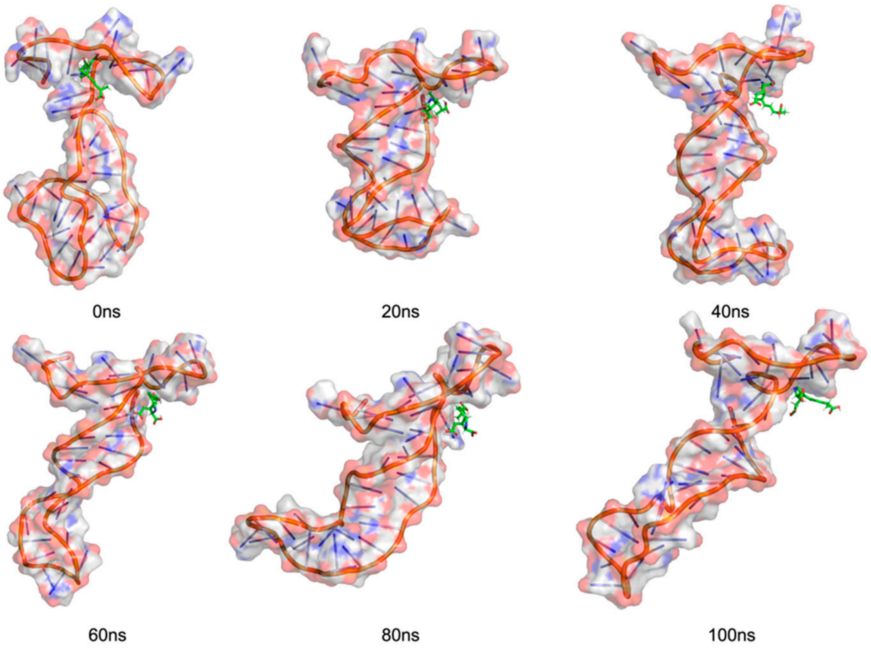

Figure S6. The structures at different simulation times of DA with aptamer C1-s. The DA was colored in green. C1-s was colored in red, white, and blue.

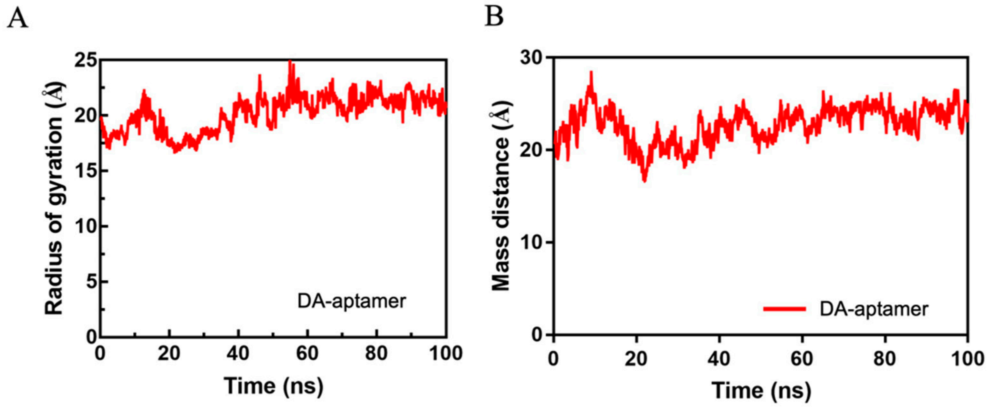

Figure S7. (**A**) Radius of gyration of complex versus simulation time. (**B**) Mass distance between DA and aptamer versus simulation time.

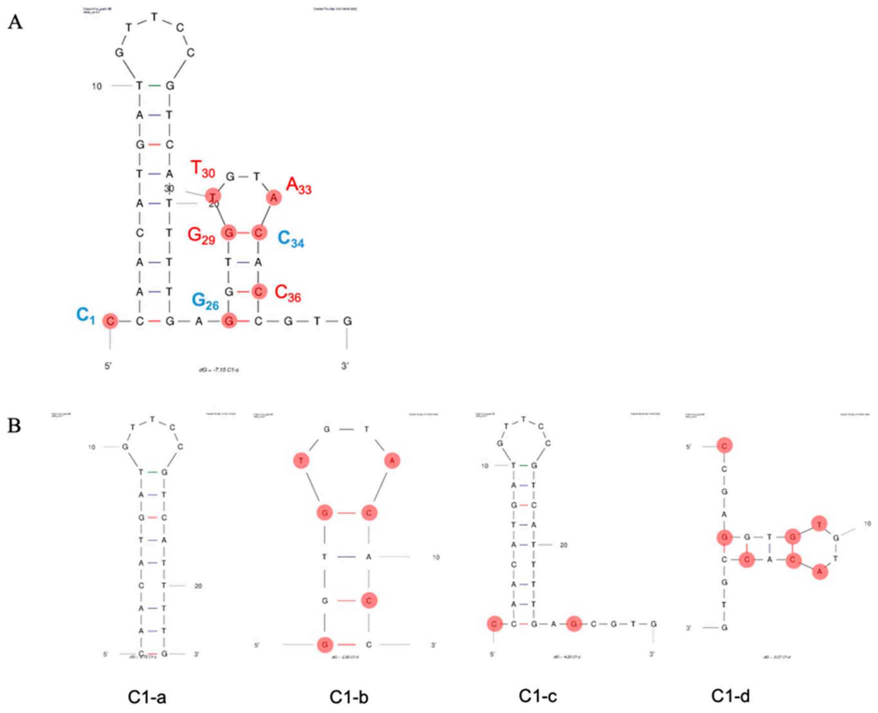

Figure S8. The positions of the key bases in the secondary structure of C1-s (**A**) and the truncated aptamers (**B**). The bases that contribute most to the complex binding free energy are marked with red circles (C_1, G_{26}, G_{29}, T_{30}, A_{33}, C_{34}, and C_{36}). And the bases marked in bold blue (C_1, G_{26}, and C_{34}) in C1-s can form hydrogen bonds with DA.

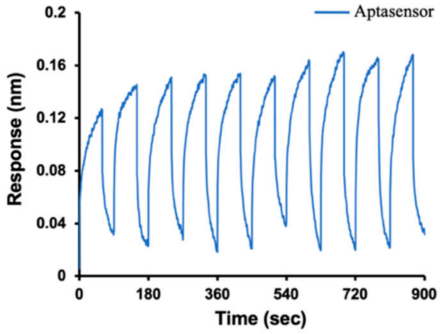

Figure S9. Assessment of the reusability of aptasensor for DA detection.

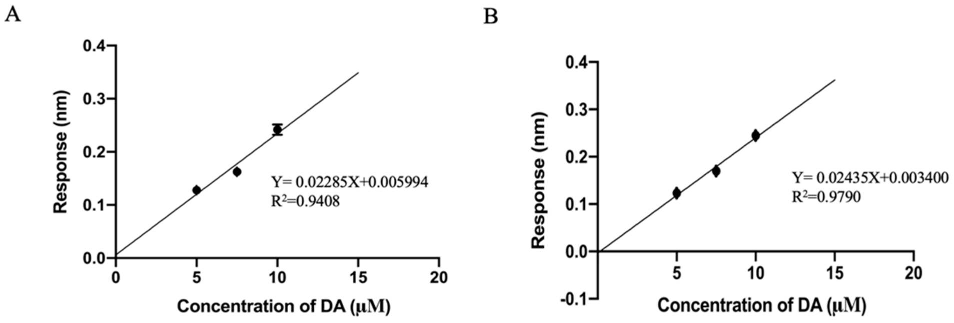

Figure S10. Standard curves in seawater (**A**) and shellfish (**B**) sample detection.

Table S1. Sequences of DNA oligonucleotides.

Name	Sequence (5'–3')
F1	ATTGGCACTCCACGCATAGG
R1	A_{20}-Spacer18-TTCACGGTAGCACGCATAGG
R2	TTCACGGTAGCACGCATAGG
Capture oligo	CCTATGCGTGGAGTGCCAAT-biotin
Library	ATTGGCACTCCACGCATAGG-N (40)-CCTATGCGTGCTACCGTGAA
Random Sequence	ATTGGCACTCCACGCATAGGATTCCGGCTAATCGACTGACTGCCGGTACG ATGCAGTCAG CCTATGCGTGCTACCGTGAA
T1	AAAGCAATTGGCACTCCACGCATAGG
T2	AACGCCATTGGCACTCCACGCATAGG
T3	AAGGCGATTGGCACTCCACGCATAGG
T4	AATGCTATTGGCACTCCACGCATAGG
T5	ACCGGCATTGGCACTCCACGCATAGG
T6	ACTGGTATTGGCACTCCACGCATAGG
T7	AGCGTCATTGGCACTCCACGCATAGG
C1-s	CCAACATGATGTTCCGTCATTTTGAGGTGTGTACACCGTG
C1-a	CAACATGATGTTCCGTCATTTTG
C1-b	GGTGTGTACACC
C1-c	CCAACATGATGTTCCGTCATTTTGAGCGTG
C1-d	CCGAGGTGTGTACACCGTG

Table S2. SELEX conditions for isolating aptamers toward DA.

Round	Amount of library (pmol)	Amount of DA (pmol)	Amount of KA (pmol)	Incubation time of DA (min)	Incubation time of KA (min)
1	2000	100	0	60	0
2	300	100	0	60	0
3	300	100	0	60	0
4	100	100	0	60	0
5	100	100	0	60	0
6	100	100	100	60	30
7	100	100	100	60	30
8	100	100	100	45	50
9	100	100	100	45	50
10	100	100	150	45	50
11	100	100	150	45	70
12	100	100	150	45	70

Table S3. The affinity of the truncated aptamers.

Name	Response (nm)	K_D (M)	K_D Error	R_2	X_2
C1-s	0.1585	1.50×10^{-6}	1.13×10^{-7}	0.9368	0.0457
C1-a	0.1267	6.90×10^{-6}	6.28×10^{-7}	0.7933	0.2485
C1-b	0.1522	1.67×10^{-7}	9.33×10^{-9}	0.9455	0.0450
C1-c	0.1290	4.90×10^{-6}	3.12×10^{-7}	0.8013	0.0819
C1-d	0.1701	1.09×10^{-7}	9.31×10^{-9}	0.9469	0.0311

Table S4. Average Binding Energy and its Components Obtained from the MM-GBSA Calculation for the DA-aptamer.

Contribution	Energy (kcal/mol)
ΔEvdw	-24.0262±0.21
ΔEele	-44.0991±0.68
ΔGpolar	57.7329±0.58
ΔGnonpolar	-3.1129±0.02
ΔGtotal	-13.5053±0.19

Table S5. Energy decomposition.

Base	ΔEvdw	ΔEele	ΔGsol	ΔGtotal
G29	-3.0546	0.676885	0.067766	-2.30995
C1	-1.72406	-1.49513	2.133066	-1.08612
C34	-2.37374	-1.80076	3.135828	-1.03868
G26	-1.32287	-12.0155	12.45581	-0.88258
C36	-0.34203	-1.99945	2.024968	-0.31651
A33	-0.26956	-0.64659	0.909442	-0.00671
T30	-0.28887	-0.06438	0.351863	-0.00138

Table S6. The response values of blank samples.

NO.	Response (nm)	NO.	Response (nm)
1	0.0004	11	0.0006
2	0.0006	12	0.0006
3	0.0005	13	0.0005
4	0.0005	14	0.0007
5	0.0006	15	0.0004
6	0.0005	16	0.0005
7	0.0005	17	0.0005
8	0.0006	18	0.0006
9	0.0004	19	0.0004
10	0.0007	20	0.0004

Table S7. Detection of DA concentrations around (or below) the theoretical LOQ.

Conc. (nM)	Response (nm)	R^2	X^2	Mean±SD	Theoretical value (nm)
	0.01282	0.86	0.1069		
50	0.01341	0.8075	0.1291	0.01294±0.00042712	0.0128
	0.01258	0.7968	0.0927		
	0.01250	0.7884	0.2273		
37.5	0.01263	0.8023	0.1470	0.01264±0.00014526	0.01271
	0.01279	0.791	0.3523		
	0.01069	0.813	0.0591		
25	0.01238	0.7792	0.1038	0.01164±0.0086435	0.01245
	0.01185	0.8387	0.0294		
